# Study of Virtual Reality Immersive Technology Enhanced Mathematics Geometry Learning

**DOI:** 10.3389/fpsyg.2022.760418

**Published:** 2022-02-17

**Authors:** Yu-Sheng Su, Hung-Wei Cheng, Chin-Feng Lai

**Affiliations:** ^1^Department of Computer Science and Engineering, National Taiwan Ocean University, Keelung, Taiwan; ^2^Department of Engineering Science, National Cheng Kung University, Tainan, Taiwan

**Keywords:** virtual reality technologies, mathematics learning aids, geometry education, immersive learning, emerging technologies

## Abstract

Mathematics is an important foundation for the development of science education. In the past, when instructors taught mathematical concepts of geometry shapes, they usually used traditional textbooks and aids to conduct teaching activities, which resulted in students not being able to understand the principles completely. Nowadays, it has become a trend to integrate emerging technologies into mathematics courses and to use digital instructional aids. Emerging technologies can effectively enhance students’ sensory experience while strengthening their impressions and understandings of subject concepts. In this paper, we apply virtual reality immersive technologies to develop a “virtual reality immersive learning mathematics geometry system,” which is used to teach mathematical geometry concepts. Teachers use the system to develop three basic mathematical geometry learning materials: “Triangular pyramid volume = 1/3 prism volume,” “Cone volume calculation,” and “Triangle center of gravity derivation.” In the experimental activity, the teacher uses virtual reality teaching aids to guide students to learn mathematical geometry concepts in a fun way so that they can achieve the effectiveness of immersive learning. This study explores the impact of using the virtual reality immersive learning mathematics geometry system on students’ technology acceptance, learning motivations, and learning performance. The experimental result showed that using the virtual reality immersive learning mathematics geometry system can improve the learning motivation and learning performance of students. The findings indicated that the experimental group had better learning outcomes after completing the learning tasks of three geometric units. The experimental group used the virtual reality immersive learning mathematics geometry system which can lead to better learning outcomes. According to the ARCS questionnaire, students in the experimental group were confident to understand new subjects. At the same time, the mode of completing the game can effectively give students a sense of accomplishment. The use of emerging technologies in the classroom can be an attractive learning mode for students.

## Introduction

Mathematics is a very important cornerstone of science education, and also a kind of regular logic science. If students are exposed to mathematics at the early age and are not excluded from the operation of mathematical concepts, it will help develop their logic. The current 12-year national education to college education in Taiwan is gradually changing and providing opportunities for students to learn and expose to mathematics practically (Merchant et al., 2014; Cai et al., 2017; Chou et al., 2020; Lai et al., 2021). However, in the past, most mathematics teachers used traditional mathematics textbooks to teach concepts of geometric shapes and conduct expository teaching activities, in which the concept of geometric shapes contains important logical concepts of space such as spatial perception, spatial visualization, mental rotation, spatial relationships, spatial orientation, etc., (Kaufmann et al., 2000; Potkonjak et al., 2016; Su and Wu, 2021). If students only accept the teaching contents in the classroom or learn the concept of geometry alone for a long time, they only learn the calculation and derivation of geometric formulas through book exercises, which lack of stimulation and variation, and thus cannot enhance the motivation of students to learn geometry actively (Karakis et al., 2016). Nowadays, Virtual Reality (VR) is an emerging technology that is gradually being used as a teaching aid for mathematics, it can make it easier for students to learn. Teachers can use VR technology to facilitate students’ learning of geometric mathematics and enhance students’ motivation to learn effectively (Wang et al., 2018).

In this study, the virtual reality immersive learning mathematics geometry system was used to complete three important geometric units learning tasks, such as “Triangular pyramid volume = 1/3 prism volume,” “Cone volume calculation,” and “Triangle center of gravity derivation.” Teachers can use these three mathematics learning aids to enhance students’ learning motivation in mathematics courses, making the abstract concept of geometric space more concrete, and at the same time, greatly enhance their conceptual thinking. We designed the pretest, posttest, and questionnaires to investigate the corresponding changes in students’ learning of mathematical geometry concepts by using virtual reality technology and the impact of using the virtual reality immersive learning mathematics geometry system on technology acceptance, learning motivation, and learning effectiveness. In this study, we propose three research questions:

- Is the experimental group having better learning outcomes after using the virtual reality immersive learning mathematics geometry system to learn and completing the three learning tasks of geometric units?
- What is the difference in the result of the ARCS questionnaire between the experimental group and the control group?
- What is the difference in the result of the TAM questionnaire between the experimental group and the control group?

## Literature Review

### Virtual Reality Immersive Technologies for Mathematics Education

The demonstration of virtual reality teaching can help students understand complex mathematical logical concepts and reduce students’ misunderstandings (Mikropoulos and Natsis, 2011). Virtual reality learning aids provide students with a stronger sense of immersion and presence. Immersion allows students to feel realistic through virtual simulations, while presence provides students with different levels of sensory experience. Students participate in a virtual reality classroom to learn geometric math, which provides a good immersive virtual learning environment (Hussein and Nätterdal, 2015). Virtual reality learning aids have important advantages over increasing students’ motivation to learn, exploring principles and visualizing abstract things, and the immersion mechanism can effectively stimulate students’ motivation to learn new knowledge (Wang et al., 2018). Guerrero et al. (2016) create a virtual reality learning environment where students can take on the role of virtual characters and change their position, size, and use the control interface to manipulate mathematical geometries learning basic mathematical concepts of geometry.

To summarize the above literature review, using virtual reality technology to assist students’ learning as a teaching aid is an innovative way to teach mathematical geometry. The diversity of virtual reality technology in any mathematical concepts education can be further promoted. Immersive technologies like augmented reality(AR), virtual reality(VR) and mixed reality(MR) in the curriculum provide many benefits in teaching. For example, it can provide a platform to increase students’ enjoyment, and give students a different learning experience (Osypova et al., 2020). Osypova et al. (2020) think that the immersive technologies in the curriculum can increase the effectiveness of learning, and enhance the motivation of students in class.

### Virtual Reality Mathematics Geometry Teaching Platform

Virtual reality mathematics geometry teaching platforms such as “ClassVR,” “VRMath,” and “GeoGebra” can be found on the market today (ClassVR, 2017; VRMath, 2017; GeoGebra, 2019). The ClassVR platform provides an operating record system that allows teachers to understand how students are learning by using the platform (ClassVR, 2017). The VRMath platform provides geometric mathematical virtual objects, such as cylinders, cones, and trigonometric cones (VRMath, 2017). GeoGebra is a three-dimensional drawing platform that provides a three-dimensional learning space, including *X*, *Y*, and *Z*-axis virtual space, in which students can add points and lines in the virtual space to create a 3D virtual object. The GeoGebra platform is more complicated to operate for beginners (GeoGebra, 2019). ClassVR, VRMath, GeoGebra, and other virtual reality technologies have their advantages and disadvantages when integrated into geometric math teaching platforms. To meet the needs of experienced mathematics teachers, this study uses virtual reality technology to develop a virtual reality immersive learning mathematics geometry system, which specifically implements three geometric 3D virtual reality and mathematics app application teaching units such as triangular cone volume = 1/3 of the triangular column volume, cone calculation, and triangle center of gravity derivation.

### ARCS Learning Motivations

The learning motivation is a major factor that affects learning. Learning is driven by motivation, with students who are highly motivated to learn showed higher learning outcomes. The teacher’s educational model of combining mathematics concepts through virtual reality emerging technologies provides teachers the opportunity to teach students with better motivation for learning, and also to understand students’ learning status. Learning Motivation allows students to become more involved in the learning situation, to feel the importance of the problem, and also be motivated to solve it (Jarmon et al., 2009; Chen, 2013; Karakis et al., 2016; Keller, 2016; Bacca et al., 2018). Wang et al. (2018) used the ARCS learning motivation model to conduct a questionnaire survey and found that using virtual reality technology for education can satisfy the ARCS learning motivation model, aroused students’ interest in learning mathematics and science and build their confidence in interactive learning, which can then be used to understand the effectiveness and satisfaction of students’ immersive learning.

This study used the ARCS learning motivation questionnaire to explore students’ motivation to learn geometry (Karakis et al., 2016; Wang et al., 2018). The ARCS learning motivation questionnaire was designed to promote student interaction and engagement in virtual reality mathematics materials. The learning motivation questionnaire is designed to address four dimensions: Attention, relevance, confidence, and satisfaction.

### Technology Acceptance Model

Technology Acceptance Model (TAM) is a model for understanding user acceptance of new technologies, developed specifically to explain or predict user acceptance of emerging computer technologies (Davis, 1989). TAM is now used as the theoretical basis for many empirical studies of user acceptance of new technologies (Davis, 1989). The purpose of the questionnaire is to understand students’ perceived usefulness and perceived ease of use related to the integration of virtual reality technology into academic teaching materials. Faizah and Nurshamshida (2019) used the TAM to know the factors which could influence the students’ acceptance of virtual reality in class. They think that virtual reality could be an appropriate platform to integrate into the classroom.

In this paper, we try to investigate whether the virtual reality technology can effectively help students’ learning in mathematics at the level of perceived usefulness, and to understand whether the experimental system is easy to use at the level of perceived ease of use.

## Virtual Reality Immersive Learning Mathematics Geometry System

**Figure 1:**
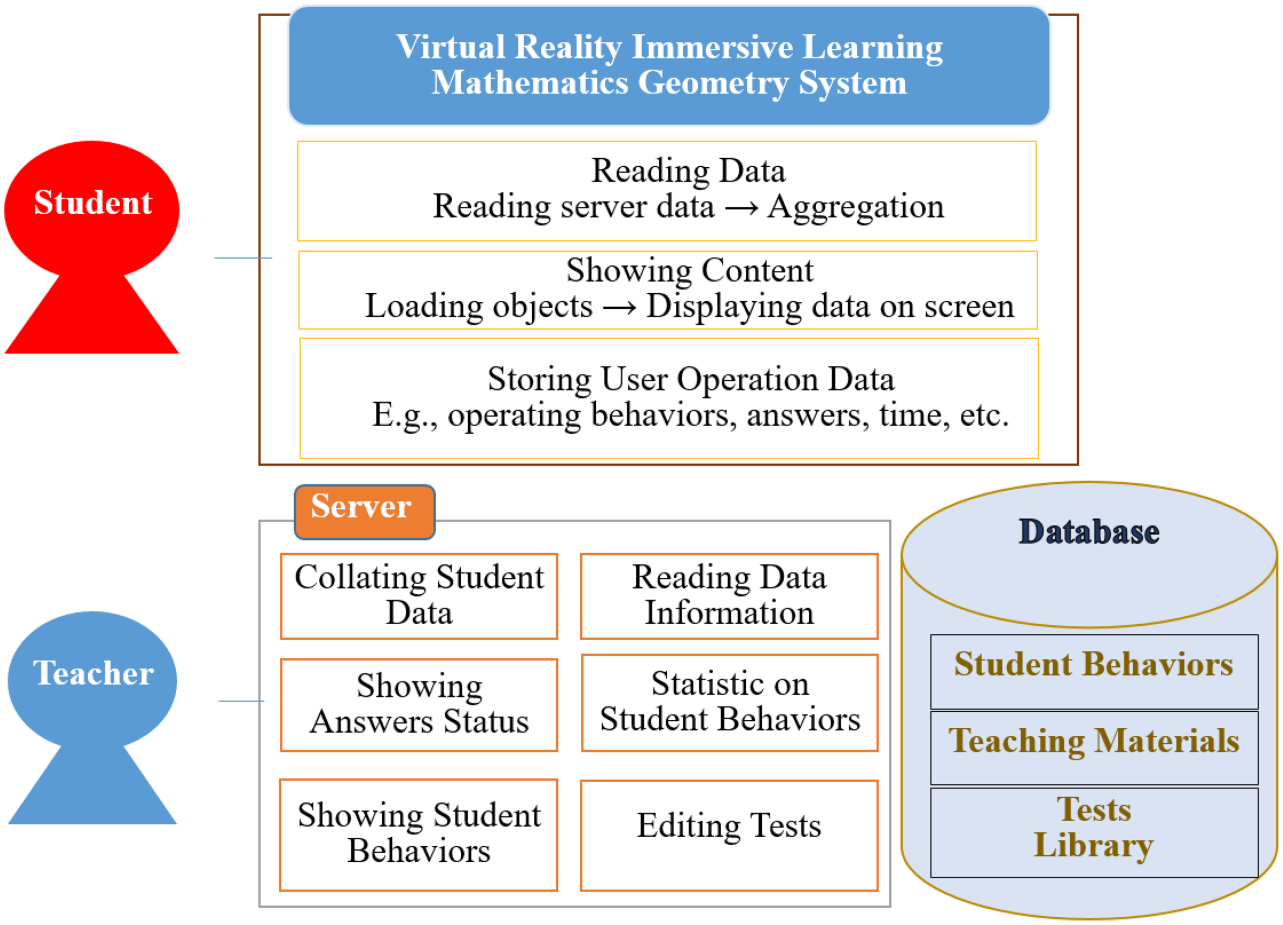
The architecture of the virtual reality immersive learning mathematics geometry system.

In this study, we use virtual reality immersive learning mathematics geometry system integrated into a geometric graphing mathematics course. There are two different roles: teacher side and student side in this virtual reality immersive learning mathematics geometry system, which is the most important feature. After entering the system, three mathematical units appear on the screen, such as “Triangular pyramid volume = 1/3 prism volume,” “Cone volume calculation,” and “Triangle center of gravity derivation.” Teachers can then compile test questions and content in these three modules, and can upload new content to the virtual reality immersive learning mathematics geometry system at any time, emphasizing that the content can be updated at any time. The generic architecture of the virtual reality immersive learning mathematics geometry system is as shown in Figure 1.

**Figure 2:**
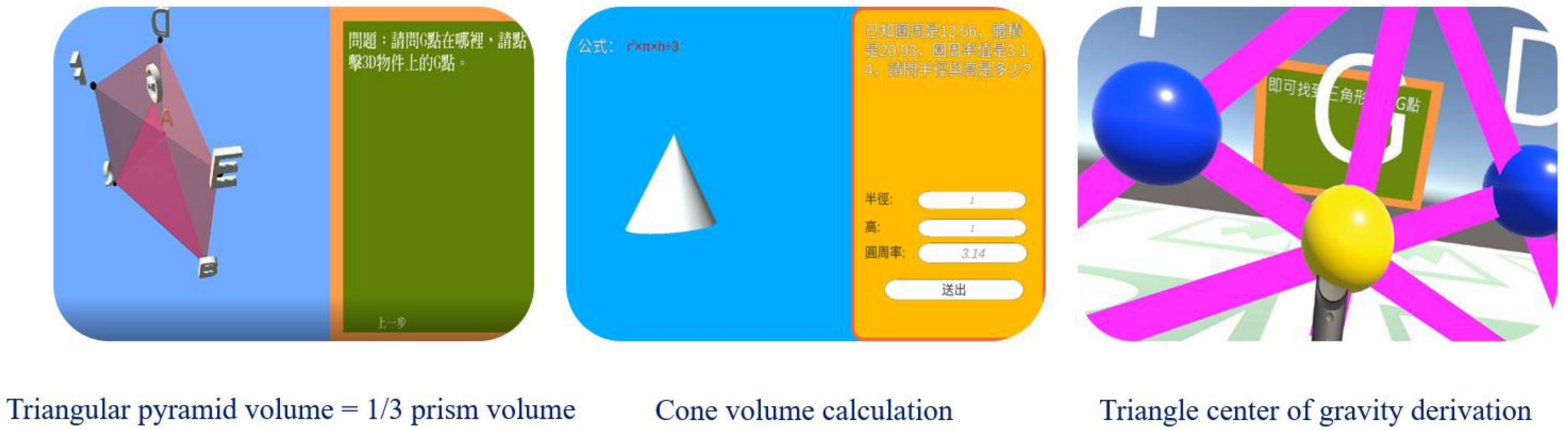
Three learning units in the virtual reality immersive learning mathematics geometry system.

In the first learning unit “Triangular pyramid volume = 1/3 prism volume,” students learn that triangular cone volume is equal to 1/3 of the triangular column volume through the system step by step. In the second learning unit “Cone volume calculation,” the teacher uses the system to teach students how to calculate the volume of a cone. In the third learning unit “Triangle center of gravity derivation,” students use 3D helmets and controllers to create a 3D virtual triangle in the virtual reality learning space, and observe the center of gravity of the triangle. Three learning units in the system are shown in Figure 2.

The novelty of the system is that it records students’ actions and responses, and then sends them to the virtual reality immersive learning mathematics geometry system, allowing teachers to know students’ learning status in real-time, focusing on the system function of immediate assessment.

## Methodology

This study is based on teachers’ past experience in teaching basic mathematical geometry knowledge, and uses the virtual reality technology to develop a virtual reality immersive learning mathematics geometry system to enhance students’ sensory experiences about mathematical geometry concepts.

### Participants

The experimental subjects were 40 students from two classes of the second grade in a high school in the northern city of Taiwan. The students were divided into two groups, one as the experimental group with 20 students, and the other as the control group with 20 students. The students in both classes were taught by the same teacher at different points in time. Students in the experimental group used the virtual reality immersive learning mathematics geometry system to learn basic mathematical geometry concepts, while teachers of the control group used traditional paper-based materials with a narrative mode of instruction to teach basic mathematical geometry concepts.

### Learning Materials

In this study, students were introduced to the knowledge and concepts of mathematical geometry by collaborating with experienced mathematics teachers and following the suggestions of mathematics teachers to select three important mathematical topics in the concept of mathematical geometry such as “Triangular pyramid volume = 1/3 prism volume,” “Cone volume calculation,” and “Triangle center of gravity derivation.” The experimental group and control group students will complete the three learning tasks through the curriculum experimental activities with different learning modes and materials.

### Procedure

**Figure 3:**
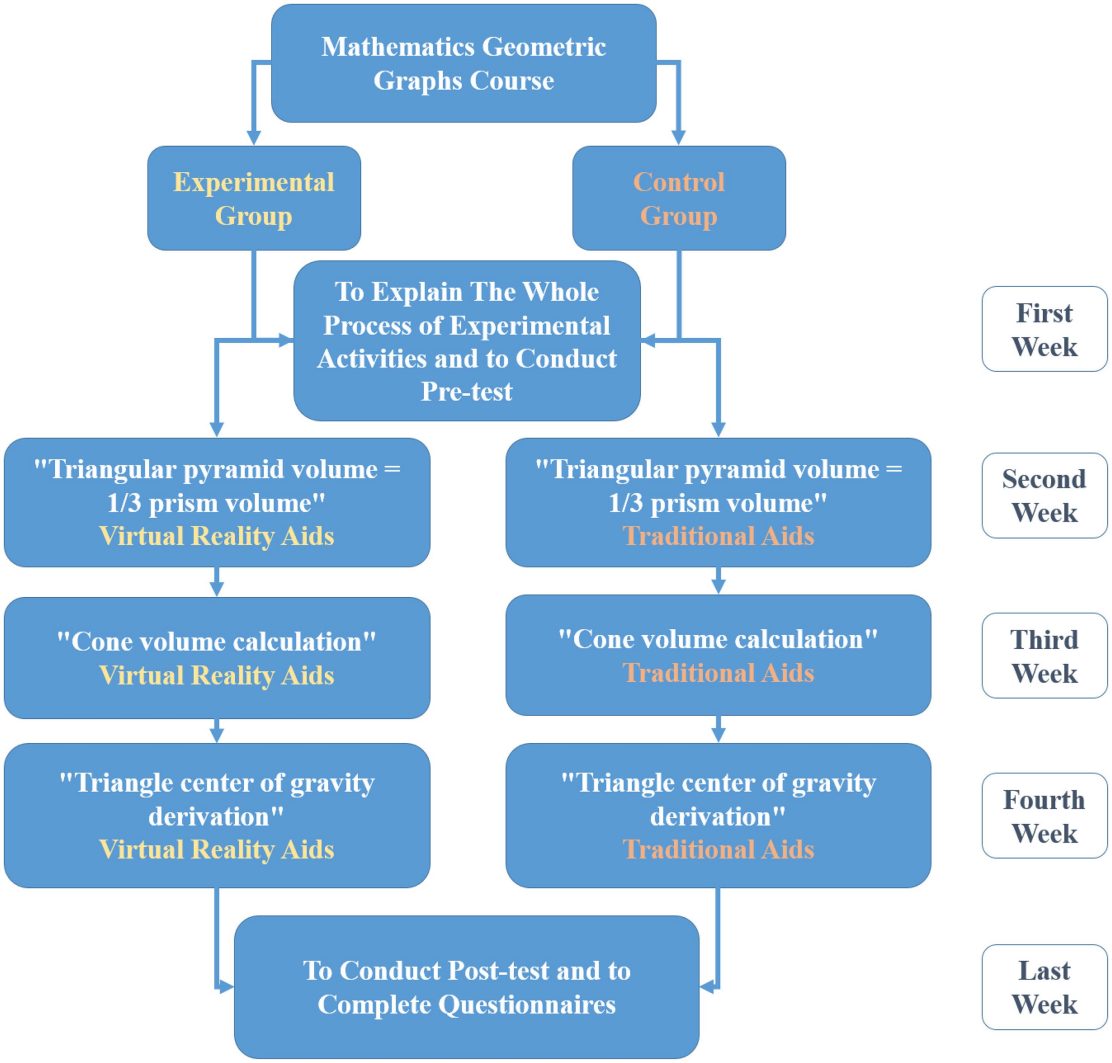
Procedure.

The entire experimental process and procedure proceed for 5 weeks. Each week, students have to complete a designated experiential learning activity, each activity lasts for 100 min, as shown in Figure 3. In the first week, each student was given a pre-test to understand the differences in their prior knowledge of basic geometric concepts in mathematics. Both groups used the same teaching method, with students in the control group using traditional paper-based teaching tools with narrative instruction, and students in the experimental group using the virtual reality immersive learning mathematics geometry system to learn. In the next three weeks, students in the control group using traditional paper-based teaching tools with narrative instruction to study, and students in the experimental group using the virtual reality immersive learning mathematics geometry system to learn three important mathematical topics in the concept of mathematical geometry such as “Triangular pyramid volume = 1/3 prism volume,” “Cone volume calculation,” and “Triangle center of gravity derivation.” In the last week, each student was given the posttest, the learning motivation questionnaire, and the TAM questionnaire. The posttest was conducted to measure students’ learning achievements. The learning motivation questionnaire surveyed the students’ learning motivation. The TAM questionnaire was conducted to know students’ acceptance of using the virtual reality immersive learning mathematics geometry system.

### Instruments

The experimental instruments used in the experimental activity of this study include the pretest and the posttest, the learning motivation questionnaire, and the TAM questionnaire.

#### Pretest and Posttest

The experimental materials for this study were obtained from the second grade mathematics textbook of Han Lin Publishing in Taiwan (Wu et al., 2019). The pretest and posttest were the same test assessment. The pretest is designed to understand students’ prior knowledge of the three learning units in the high school mathematics curriculum, such as “Triangular pyramid volume = 1/3 prism volume,” “Cone volume calculation,” and “Triangle center of gravity derivation,” while the post-test measures students’ learning effectiveness. The test consists of 20 multiple-choice questions (5 points each), totaling 100 points. The test was predicted by 40 students and analyzed by the IBM SPSS statistical tool to verify the discrimination and difficulty of the test. The degree of discrimination is 42%, and the degree of difficulty is 45%.

#### Learning Motivation Questionnaire

In this study, we refer to the ARCS learning motivation questionnaire from the previous studies and modified it into the learning motivation questionnaire in this paper (Karakis et al., 2016; Wang et al., 2018). There are 24 items on a 5-point Likert scale in this questionnaire. The motivation questionnaire of this experiment contains four dimensions of ARCS, such as Attention, Relevance, Confidence, and Satisfaction. There are six questions for each of these dimensions. The motivation questionnaire was pre-tested by 40 students and the reliability was assessed by using the IBM SPSS statistical tool. The Cronbach alpha coefficients were 0.73 for attention, 0.75 for relevance, 0.78 for confidence, and 0.80 for satisfaction. Four dimensions are found to be reliable.

#### Technology Acceptance Model

In this study, the technology acceptance model questionnaire was modified from the previous studies (Chang et al., 2011). The questionnaire has 16 questions using a 5-point Likert scale. This questionnaire was pre-tested by 40 students and the validity of the technology acceptance questionnaire was evaluated by using IBM SPSS statistical tool. The Cronbach alpha coefficient was 0.81, meaning that the questionnaire has internal consistency and reliable reliability.

## Results

### Analysis of Prior Knowledge and Learning Effectiveness

According to Table 1, the analysis of the pretest, the mean of the experimental group was 33.750 with a standard deviation of 14.130, while the mean of the control group was 32.250 with a standard deviation of 15.684. From the low mean of both groups, it can be seen that most of the students were not familiar with the content of the mathematics subject before the course.

**Table 1:** Analysis of the independent sample *t*-test of the pretest of two groups.

Group	*N*	Mean	SD	*t*	Sig.
Experimental group	20	58.250	10.036	1.905	0.369
Control group	20	51.500	12.258		

*p < 0.05.

The posttest was conducted after the two groups of students had completed the experimental activities of course learning. According to Table 2, the analysis of the post-test data, the mean of the experimental group was 58.250 with a standard deviation of 10.036, while the mean of the control group was 51.500 with a standard deviation of 12.258, and *t*-value is 1.905. This result showed that the experimental group students had significantly higher posttest scores than the control group students. In this experimental study, the mean score of the experimental group was higher than the control group (58.250 > 51.500) according to the post-test results, and the difference in standard deviation between the two groups was not significant.

**Table 2:** Analysis of the independent sample *t*-test of the posttest of two groups.

Group	*N*	Mean	SD	*t*	Sig.
Experimental group	20	33.750	14.130	0.318	0.619
Control group	20	32.250	15.684		

*p < 0.05.

### Analysis of the Learning Motivation

The learning motivation questionnaire was designed to investigate students’ motivation for learning mathematics concepts. According to Table 3, the mean of the experimental group was 3.875 with a standard deviation of 0.718. And the mean of the control group was 3.488 with a standard deviation of 0.711, the mean of the experimental group was higher than the mean of the control group, the result showed that the mean data of the experimental group was better than the control group (3.875 > 3.488). The effect size is used to detect the effect of the independent sample t-test (Nakagawa and Cuthill, 2007), where Cohen’s d value is most commonly used as a criterion for evaluation. Cohen’s d value evaluation criteria are small effects (≥0.2 and <0.5), medium effect (≥0.5 and <0.8), and large effect (≥0.8; Cohen, 1988). The results of this study showed that the experimental group had better learning motivation analysis results than the control group, where the Cohen’s value of the independent sample t-test fell in the medium effect range. Most of the students reported that the virtual reality immersive learning mathematics geometry system using headset 3D helmets to make the learning process fun for the experimental group, and it was easier for the experimental group to focus on their learning, which was also the key to catch students’ attention (Huang and Chen, 2019). In both two experiments, the experimental group had better learning motivation than the control group. The experimental group data showed that students were enthusiastic, curious, and novel about the virtual reality learning materials (Yen, 2017).

**Table 3:** Analysis of the learning motivation of two groups.

Group	*N*	Mean	SD	*t*	Sig.
Experimental group	20	3.875	0.718	3.423	0.001^**^
Control group	20	3.488	0.711		

** *p < 0.01*.

### Analysis of the Technology Acceptance Model

According to Table 4, from the analytical result of the technology acceptance model questionnaire, the mean value of the experimental group was 3.875 with a standard deviation of 0.7906 and the mean value of the control group was 3.750 with a standard deviation of 0.7425. The mean value of the experimental group study results was higher than the control group (3.875 > 3.750).

**Table 4:** Analysis of the technology acceptance model of two groups.

Group	*N*	Mean	SD	*t*	Sig.
Experimental group	20	3.875	0.7906	1.152	0.256
Control group	20	3.750	0.7425		

*p < 0.05.

In terms of perceived usefulness, the use of the virtual reality immersive learning mathematics geometry system with hardware devices such as helmets and joysticks for educational purposes can help students understand abstract mathematical concepts and derivation processes more quickly. In terms of perceived ease of use, the experiment provided an additional instruction manual for the operation of the virtual reality immersive learning mathematics geometry system so that students could become familiar with the use of the virtual reality system as soon as possible. According to the analysis of the independent sample t-test, the results showed that the technology acceptance results of the experimental group were slightly better than those of the control group (*t* = 1.152). According to the results of previous studies, it was found that, perceived usefulness and perceived ease of use can have a significant impact on learners’ intentions to use the virtual reality immersive learning mathematics geometry system for education (Huang and Liaw, 2018).

## Conclusion

In this paper, we integrate virtual reality technology into mathematics education content, using virtual reality technology to integrate mathematical geometry concepts into the content, and also allowing students to use the virtual reality immersive learning mathematics geometry system. Students can immerse themselves into the content learning process of the mathematics subject. It is expected that students will be motivated to take the initiative to learn mathematics, and also expected to inspire students to take the initiative to learn mathematics. The course experimental activities contain three important mathematical geometry learning units such as “Triangular cone volume = 1/3 of the angular column volume,” “Cone volume calculation,” “Triangle center of gravity derivation,” through the virtual reality of the 3D head-mounted display, App teaching application to complete the above three geometric basic concepts of learning. Students will be able to carry out learning tasks and acquire all important knowledge of basic mathematical geometry through the course activities. The primary goal is to provide students with a solid understanding of the core concepts of geometric graphs in the mathematics subject. According to the results, there are some findings we have found. The experimental group had better learning outcomes after completing the learning tasks of three geometric units. The experimental group used the virtual reality immersive learning mathematics geometry system to lead to better learning outcomes. According to the ARCS questionnaire, students in the experimental group were confident to understand new subjects. At the same time, the mode of completing the game can effectively give students a sense of accomplishment. The use of emerging technologies in the classroom can be an attractive learning mode for students.

## Data Availability Statement

The original contributions presented in the study are included in the article/supplementary material, further inquiries can be directed to the corresponding author.

## Ethics Statement

Ethical review and approval was not required for the study on human participants in accordance with the local legislation and institutional requirements. Written informed consent for participation was not required for this study in accordance with the national legislation and the institutional requirements.

## Author Contributions

Y-SS, H-WC, and C-FL contributed equally to the conception of the idea, implementing and analyzing the experimental results, and writing the manuscript. All authors read and approved the final manuscript.

## Funding

This study was supported by the Ministry of Science and Technology of Taiwan under contract numbers MOST 109-2511-H-019-004-MY2 and MOST 109-2511-H-019-001.

## Conflict of Interest

The authors declare that the research was conducted in the absence of any commercial or financial relationships that could be construed as a potential conflict of interest.

## Publisher’s Note

All claims expressed in this article are solely those of the authors and do not necessarily represent those of their affiliated organizations, or those of the publisher, the editors and the reviewers. Any product that may be evaluated in this article, or claim that may be made by its manufacturer, is not guaranteed or endorsed by the publisher.
